# Optimization of a cisplatin model of chemotherapy-induced peripheral neuropathy in mice: use of vitamin C and sodium bicarbonate pretreatments to reduce nephrotoxicity and improve animal health status

**DOI:** 10.1186/1744-8069-10-56

**Published:** 2014-09-04

**Authors:** Josée Guindon, Liting Deng, Baochang Fan, Jim Wager-Miller, Andrea G Hohmann

**Affiliations:** Department of Psychological and Brain Sciences, Gill Center for Biomolecular Science, Indiana University, 1101 E. 10th St, Bloomington, IN 47405-2204 USA; Department of Molecular and Cellular Biochemistry, Interdisciplinary Biochemistry Graduate Program, Indiana University, Bloomington, IN USA; Program in Neuroscience, Indiana University, Bloomington, IN USA

**Keywords:** Chemotherapy, Cisplatin, Neuropathic pain, Creatinine, NaHCO_3_ (sodium bicarbonate), Vitamin C, Resveratrol, Morphine, Ibuprofen, Interleukins (IL-6, IL-1β, IL-10, TNF-α)

## Abstract

**Background:**

Cisplatin, a platinum-derived chemotherapeutic agent, produces antineoplastic effects coupled with toxic neuropathic pain and impaired general health status. These side-effects complicate long term studies of neuropathy or analgesic interventions in animals. We recently demonstrated that pretreatment with sodium bicarbonate (4% NaHCO_3_) prior to cisplatin (3 mg/kg i.p. weekly up to 5 weeks) was associated with improved health status (i.e. normal weight gain, body temperature, creatinine and ketone levels, and kidney weight ratio) in rats (Neurosci Lett 544:41-46, 2013). To reduce the nephrotoxic effects of cisplatin treatment in mice, we compared effects of sodium bicarbonate (4% NaHCO_3_ s.c_._), vitamin C (25 mg/kg s.c_._), resveratrol (25 mg/kg s.c_._) and saline (0.9% NaCl) pretreatment on cisplatin-induced changes in animal health status, neuropathic pain and proinflammatory cytokine levels in spinal cord and kidney.

**Results:**

Cisplatin-treated mice receiving saline pretreatment exhibited elevated ketone, creatinine and kidney weight ratios, representative of nephrotoxicity. Vitamin C and sodium bicarbonate lowered creatinine/ketone levels and kidney weight ratio whereas resveratrol normalized creatinine levels and kidney weight ratios similar to saline pretreatment. All pretreatments were associated with decreased ketone levels compared to saline pretreatment. Cisplatin-induced neuropathy (i.e. mechanical and cold allodynia) developed equivalently in all pretreatment groups and was similarly reversed by either morphine (6 mg/kg i.p.) or ibuprofen (6 mg/kg i.p.) treatment. RT-PCR showed that mRNA levels for IL-1β were increased in lumbar spinal cord of cisplatin-treated groups pretreated with either saline, NaHCO_3_ or resveratrol/cisplatin-treated groups. However, IL-6 and TNF-alpha were elevated in the kidneys in all cisplatin-treated groups. Our studies also demonstrate that 60 days after the last cisplatin treatment, body weight, body temperature, kidney functions and mRNA levels have returned to baseline although the neuropathic pain (mechanical and cold) is maintained.

**Conclusions:**

Studies employing cisplatin should include NaHCO_3_ or vitamin C pretreatment to improve animal health status and reduce nephrotoxicity (lower creatinine and kidney weight ratio) without affecting the development of chemotherapy-induced neuropathy or analgesic efficacy.

## Background

Neck cancer patients receive cycles of the platinum-derived chemotherapeutic agent cisplatin once every four weeks over an extended and defined period of time [1, 2]. However, preclinical studies typically administer high doses of cisplatin either acutely or over restricted intervals in the context of restricted survival times [3, 4]. The failure of preclinical studies to more closely represent the clinical dosing schedule likely reflects the fact that cisplatin treatment is associated with known toxicities that impair general health status. These toxicities render long term evaluations of therapeutic efficacy and even animal survival problematic. Only a few preclinical studies have used these paradigms [5–10]. Specifically, renal toxicity [11–13] critically impairs animal health and undermines long term studies aimed at identifying mechanisms of cisplatin-induced neuropathies or effective treatments. Indeed, cisplatin produces painful as well as nonpainful sensory neuropathies through mechanisms that remain poorly understood. Cisplatin-induced nephrotoxic effects impair animal health and impede investigations aimed at understanding the resulting neuropathies and long term treatments [14–17]. Attempts to buffer acidic effects of cisplatin to minimize renal toxicity with alkaline pretreatments show beneficial effects in preclinical [8] and clinical [18] studies. Manipulating diets of cancer patients toward alkaline pH attenuates cancer cell survival and reduces renal toxicity, a dose-limiting consequence of repeated cisplatin treatment [18, 19]. Human studies have demonstrated the beneficial effects of sodium bicarbonate in reducing blood acidosis and kidney toxicity in chemotherapy patients [18, 19]. Recently, we established a rat model of cisplatin-induced neuropathy in which pretreatment with sodium bicarbonate (4% NaHCO_3_ administered subcutaneously (s.c.)) prior to once weekly cisplatin dosing over 16 or 28 days minimized damage to renal functions (creatinine levels, kidney weight ratio and pH of urine). This pretreatment was associated with improved general health status (normal weight gain, normal body temperature and no mortality) that should permit long term evaluations in preclinical studies [8]. Thus, alkaline solutions can be used to counteract acidic effects of cisplatin that cause nephrotoxicity and mortality in rats. However, antioxidants such as vitamin C and resveratrol also confer protective effects against cisplatin-induced nephrotoxicity without negativetly affecting its bioavailability in rodents [13, 20–23]. This antioxidant effect is mediated by reducing oxidative stress which is protective against various injuries [21]. Moreover, vitamin C also attenuates the lipid peroxidation glutathione depletion and decreases in glomerular filtration rate that are induced by cisplatin treatment [21, 22]. Extension of these renal protective effects of alkaline diet/solution or antioxidants (vitamin C, resveratrol) to a mouse model of cisplatin-induced neuropathy is critical if we are to fully exploit the power of transgenic approaches for identifying and validating therapeutic targets for treating and preventing neuropathy in humans. The present study was designed to develop an improved mouse model of cisplatin-induced neuropathy by comparing different pretreatments (saline, sodium bicarbonate, vitamin C, or resveratrol) aimed at minimizing detrimental effects of cisplatin on body weight, body temperature, and kidney functions (creatinine levels, kidney weight ratio and urinary pH). We also evaluated transcriptional regulation of pro-inflammatory cytokines (IL-1β, IL-6, IL-10 and TNFα) in kidney and lumbar spinal cord following each treatment. These studies used a repeated cisplatin dosing paradigm to better mimic the clinical condition. We also investigated the impact of different pretreatments (i.e. saline, sodium bicarbonate, vitamin C or resveratrol) on cisplatin-induced mechanical and cold allodynia and compared the antinociceptive efficacy of reference analgesics (i.e. an opioid (morphine) and a nonsteroidal anti-inflammatory drug (ibuprofen)) since these compounds are widely use in cancer patients treated with chemotherapeutic agents [1, 2, 11, 12]. Finally, we evaluated the long term effects (60 days following cessation of repeated cisplatin dosing) of saline, saline/cisplatin and vitamin C/cisplatin-treated mice on animal health status (i.e. body weight, body temperature), kidney functions (creatinine, kidney weight ratios), neuropathic pain (mechanical and cold allodynia) as well mRNA levels of inflammatory cytokines (IL-1β, IL-6, IL-10 and TNFα) in both lumbar spinal cord and kidney.

## Results

### Cisplatin-untreated control groups

No differences were observed between any of the pretreatment groups in animals that received saline vehicle in lieu of cisplatin in body weight (F_3,14_ = 0.42, *P* = 0.739), body temperature (F_3,14_ = 1.30, *P* = 0.312), mechanical threshold (F_3,14_ = 1.03, *P* = 0.408) or latency to respond to cold stimulation (F_3,14_ = 2.20, *P* = 0.133). Similarly, no differences in blood creatinine (F_3,8_ = 0.50, *P* = 0.691), ketone (F_3,8_ = 2.62, *P* = 0.123), glucose (F_3,8_ = 0.07, *P* = 0.973) or kidney weight ratios (F_3,8_ = 0.67, *P* = 0.594) were observed in any pretreatment group not receiving cisplatin. Similarly, urine pH (F_3,8_ = 0.32, *P* = 0.813) and blood pH (F_3,8_ = 1.04, *P* = 0.425) were similar in saline/saline or pretreated (vitamin C, resveratrol or NaHCO_3_)/saline groups. Consequently, these groups were pooled into a single control group (the control/saline group) for each survival time for further statistical analysis.

### Body weight

**Figure 1:**
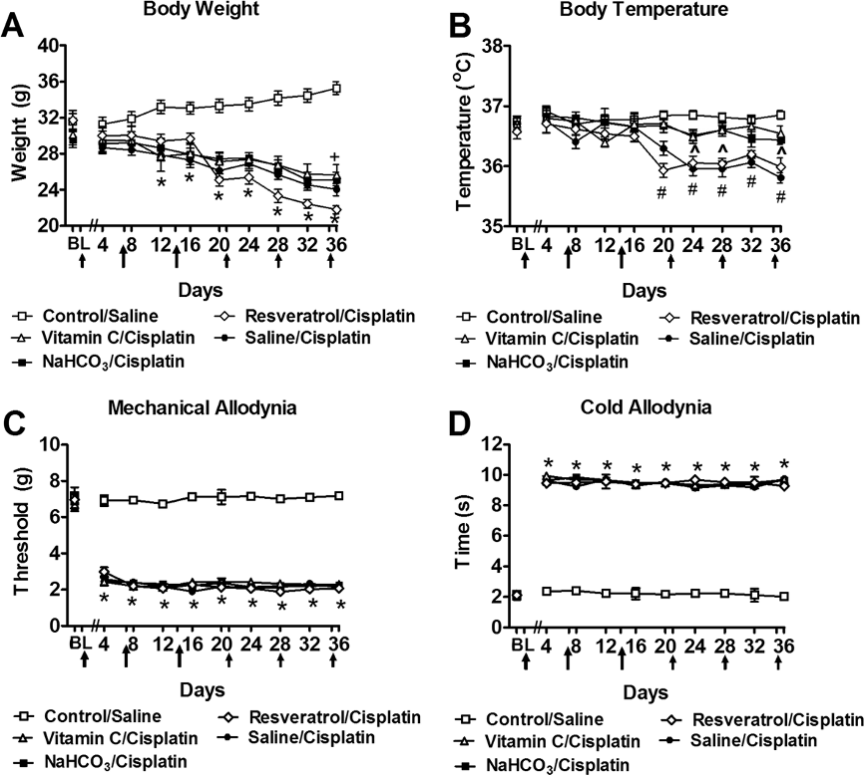
**Impact of cisplatin on body weight, body temperature, mechanical and cold allodynia in groups pretreated with antioxidant treatments (NaHCO**_**3**_ **, vitamin C, resveratrol) or saline. (A)** Body weight and **(B)** body temperature did not differ between groups receiving saline in lieu of cisplatin with different (saline, NaHCO_3_, vitamin C, resveratrol) pretreatments and were pooled into a single control/saline group. **(A)** Body weight and **(B)** body temperature was lowest in cisplatin-treated rats receiving saline or resveratrol pretreatment. Vitamin C and NaHCO_3_ attenuated cisplatin-induced decreases in body temperature. Time course of development of **(C)** mechanical and **(D)** cold allodynia in cisplatin-treated groups pretreated with saline, NaHCO_3_, vitamin C or resveratrol. Mechanical and cold sensitivity did not differ between groups receiving saline in lieu of cisplatin with different (saline, NaHCO_3_, vitamin C, resveratrol) pretreatments and were pooled into a single control/saline group. Arrows show timing of injections of chemotherapeutic agents. Data are expressed as mean ± s.e.m. (n = 9–18 per group). *P < 0.0001 for saline, NaHCO_3_, vitamin C or resveratrol/cisplatin-treated groups vs. control saline group (ANOVA, Bonferroni post hoc); + *P* < 0.048 for vitamin C vs. resveratrol/cisplatin group (ANOVA, Bonferroni post hoc); # *P* < 0.0001 for saline or resveratrol/cisplatin-treated groups vs. control saline group (ANOVA, Bonferroni post hoc); ^ *P* < 0.007 for vitamin C or NaHCO_3_/cisplatin groups vs. saline/cisplatin group (ANOVA, Bonferroni post hoc).

Body weight did not differ between any pretreatment (saline, NaHCO_3_, vitamin C, resveratrol) groups over the first 8 days of evaluation (*P* ≥ 0.114 for each observation day; Figure 1A). By contrast, the control/saline group exhibited time-dependent increases in body weight (F_36,486_ = 11.51, *P* < 0.0001; Figure 1A) from day 12 to day 36 (*P* < 0.0001 for all time points). Body weight was higher in control/saline compared to all cisplatin groups receiving different pre-treatments (F_4,54_ = 18.90, *P* < 0.0001). Indeed, weight gain in the control/saline group appeared on day 12 and persisted to day 36 (*P* < 0.042) (Figure 1A). There were no differences in body weight in the different pre-treatment/cisplatin groups from day 12 to day 32 (*P* > 0.067). However, on day 36, reseveratrol pretreatment was associated with decreased body weight in comparison to vitamin C pretreatment (*P* < 0.048).

### Body temperature

Body temperature did not differ in control/saline mice or in the different cisplatin pretreatment groups (saline, NaHCO_3_, vitamin C, resveratrol)/cisplatin-treated mice) from day 0 (Baseline) through day 16 (*P* ≥ 0.153 for each observation day; Figure 1B). However, both saline and resveratrol/cisplatin-treated groups exhibited lower body temperature relative to the control/saline group; lower body temperature was observed starting on day 20 and was maintained throughout the study (F_4,54_ = 23.75, *P* < 0.0001; days 20–36 (*P* < 0.002); Figure 1B). Body temperature did not differ in the saline/cisplatin group from that observed in the resveratrol/cisplatin group (*P* = 0.237) at any observation interval. Body temperature was lower in the saline/cisplatin groups compared to either vitamin C or NaHCO_3_/cisplatin-treatment, on day 24, 28 and 36 (*P* ≤ 0.007, *P* ≤ 0.0001 and *P* ≤ 0.0001 for each observation day, respectively). On those days, body temperature did not differ in either vitamin C/cisplatin or NaHCO_3_/cisplatin groups (*P* = 1.000).

### Cisplatin-induced mechanical and cold allodynia

Cisplatin lowered paw withdrawal thresholds in all pretreatment groups (i.e. saline, NaHCO_3_, vitamin C, resveratrol)/cisplatin-treated groups) relative to the control/saline group (F_4,54_ = 926.83, *P* < 0.0001) (Figure 1C), consistent with the development of mechanical allodynia. Mechanical allodynia was present from day 4 to day 36 (*P* < 0.0001) (Figure 1C) post initial cisplatin dosing. Furthermore, all cisplatin-treated groups showed similar increases in the latency of paw withdrawal to acetone in comparison to control/saline group (F_4,54_ = 6161.90, *P* < 0.0001) (Figure 1D), consistent with development of cold allodynia. Cold allodynia was similarly present from day 4 to day 36 (*P* < 0.0001) (Figure 1D) post initial cisplatin dosing.

### Comparison of antinociceptive efficacy of morphine and ibuprofen in cisplatin-treated mice receiving different pretreatments

**Figure 2:**
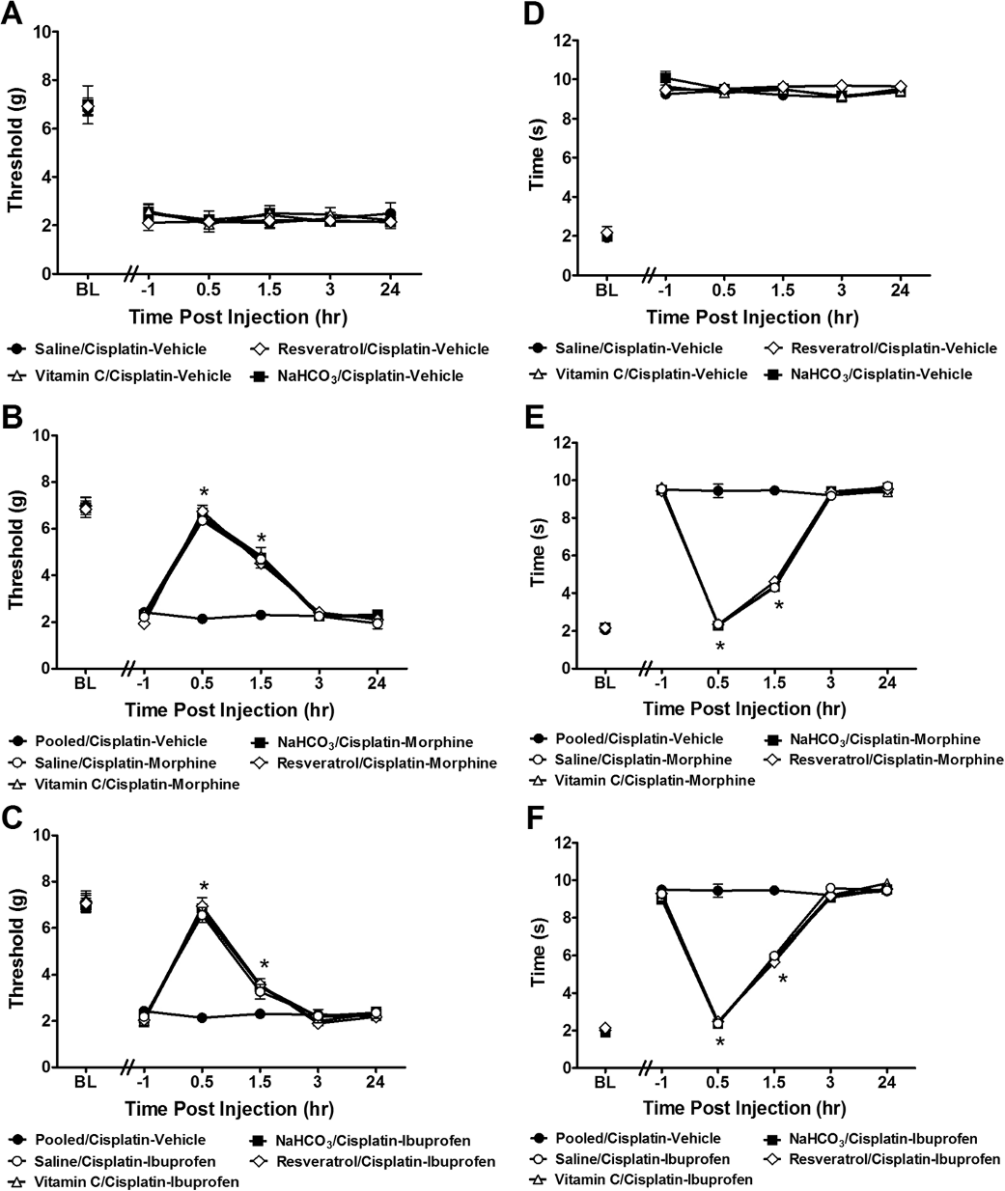
**Morphine and ibuprofen suppress cisplatin-induced mechanical and cold allodynia with equivalent efficacy in mice pretreated with saline, vitamin C, NaHCO**_**3**_ **or resveratarol.** Mechanical **(A)** and cold **(D)** sensitivity did not differ between cisplatin-treated groups receiving different pretreatments (saline, NaHCO_3,_ vitamin C, or resveratrol) that subsequently received vehicle; these groups were pooled into a single pooled/cisplatin-vehicle group. On day 36, morphine (6 mg/kg i.p.) **(B**, **E)** and ibuprofen (6 mg/kg i.p.) **(C**, **F**) produced time-dependent suppressions of cisplatin-induced mechanical **(B**, **C)** and cold **(E**, **F)** allodynia. Data are expressed as mean ± s.e.m. (n = 5 per group). *P < 0.0001 for all different (saline, vitamin C, resveratrol or NaHCO_3_) pretreated/cisplatin groups vs. control/cisplatin-vehicle group (ANOVA, Bonferroni post hoc).

There were no differences in mechanical (F_3,16_ = 0.33, *P* = 0.799; at all observation intervals *P* > 0.669; Figure 2A) or cold (F_3,16_ = 2.39, *P* = 0.107; at any time points *P* > 0.258; Figure 2D) allodynia in cisplatin-treated groups receiving vehicle that also received saline, vitamin C, resveratrol or NaHCO_3_ pretreatments_._ Therefore, these groups were pooled together into a single cisplatin-vehicle group for further statistical analyses.

**Figure 3:**
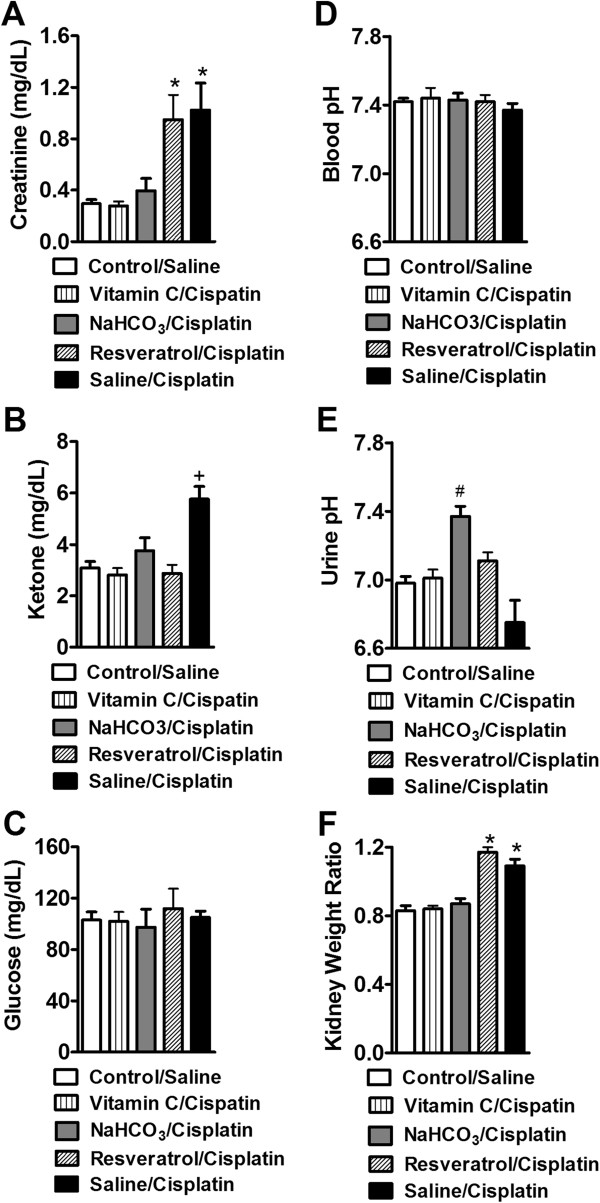
**Kidney functions assessment in saline or cisplatin-treated groups receiving different pretreatments (saline, NaHCO**_**3,**_ **vitamin C, or resveratrol). (A)** Creatinine levels were increased in saline/cisplatin and resveratrol/cisplatin groups relative to control/saline treatment. Both vitamin C and NaHCO_3_ pretreatments blocked cisplatin-induced increases in creatinine levels. **(B)** Ketone levels are increased in saline/cisplatin group but not in control/saline, vitamin C, NaHCO_3_ or resveratrol/cisplatin-treated groups. **(C)** Glucose levels and **(D)** blood pH did not differ between control/saline or any cisplatin-treated group. **(E)** Urine pH was elevated in the NaHCO_3_/cisplatin group in comparison to control/saline, saline/cisplatin or vitamin C/cisplatin groups. **(F)** Kidney weight ratios are increased in saline/cisplatin and resveratrol/cisplatin groups relative to control/saline, vitamin C or NaHCO_3_ treated groups. Data are expressed as mean ± s.e.m. (n = 8 per group). *P < 0.001 vs. control/saline, vitamin C or NaHCO3/cisplatin groups (ANOVA, Bonferroni post hoc); + *P* < 0.0001 vs. control/saline, vitamin C, NaHCO_3_ or resveratrol/cisplatin-treated groups (ANOVA, Bonferroni post hoc); # *P* < 0.0001 vs. control saline, saline/cisplatin or vitamin C/cisplatin groups (ANOVA, Bonferroni post hoc).

Morphine (6 mg/kg) suppressed cisplatin-induced mechanical (F_4,35_ = 44.33, *P* < 0.0001; Figure 2B) and cold (F_4,35_ = 849.36, *P* < 0.0001; Figure 3E) allodynia in all pretreatment (saline, NaHCO_3_, vitamin C, resveratrol; *P* < 0.0001 for each) groups relative to vehicle treatment. Morphine also produced time-dependent attenuations of mechanical (F_20,175_ = 25.76, *P* < 0.0001) (Figure 2B) and cold (F_20,175_ = 368.29, *P* < 0.0001) (Figure 2E) allodynia relative to pre-injection baseline thresholds. Anti-allodynic effects of morphine on mechanical and cold sensitivity were observed relative to vehicle at 30 (*P* < 0.0001) and 90 min (*P* < 0.0001) post-injection for each pretreatment (Figure 2B and E, respectively).

Similarly, ibuprofen (6 mg/kg) suppressed cisplatin-induced mechanical (F_4,35_ = 34.21, *P* < 0.0001; *P* < 0.0001; Figure 2C) and cold (F_4,35_ = 445.09, *P* < 0.0001; Figure 2 F) allodynia in all pretreatment (saline, NaHCO_3_, vitamin C, resveratrol) groups relative to cisplatin-vehicle treatment (*P* < 0.0001 for each pretreatment). Ibuprofen produced a time-dependent attenuation of mechanical (F_20,175_ = 22.80, *P* < 0.0001; Figure 2C) and cold (F_20,175_ = 253.99, *P* < 0.0001; Figure 2 F) allodynia relative to pre-injection baseline thresholds. Anti-allodynic effects of ibuprofen on mechanical and cold sensitivity were observed, relative to vehicle treatment, at 30 (*P* < 0.0001) and 90 min (*P* < 0.0001) post-injection in each pretreatment group (Figure 2C and F).

### Kidney functions

Both saline/cisplatin and resveratrol/cisplatin-treated groups exhibited similar (*P* = 1.000) increases in creatinine (F_4,39_ = 8.59, *P* < 0.0001) (Figure 3A) levels in whole blood relative to control/saline, vitamin C or NaHCO_3_/cisplatin-treated groups. Moreover, vitamin C and NaHCO_3_/cisplatin groups showed creatinine levels that are similar to those observed in control/saline groups (*P* = 1.000). Saline/cisplatin-treated groups also showed elevated ketone levels relative to all other treatments (F_4,39_ = 10.54, *P* < 0.0001) (Figure 3B). Blood glucose levels (F_4,39_ = 0.25, *P* = 0.906) did not differ between control/saline and any cisplatin-treated groups (Figure 3C).

No changes were observed in blood pH (F_4,39_ = 0.37, *P* = 0.829, 36 days) (Figure 3D) in any group. NaHCO_3_/cisplatin treatment (F_4,39_ = 9.57, *P* < 0.0001) (Figure 3E) increased urinary pH in comparison to control/saline, saline/cisplatin or vitamin C/cisplatin-treated groups (Figure 3E). There was no difference in the urinary pH of these latter groups (control saline, saline/cisplatin or vitamin C/cisplatin) (*P* > 0.158). NaHCO_3_/cisplatin increased urinary pH to a level comparable to resveratrol pretreatment (*P* > 0.149) (Figure 3E). Resveratrol/cisplatin and saline/cisplatin groups also exhibited elevated kidney weight ratios (F_4,39_ = 24.99, *P* < 0.0001) 36 days post initial cisplatin dosing relative to either control/saline, vitamin C or NaHCO_3_/cisplatin-treated groups (Figure 3 F). The kidney weight ratio of control/saline, vitamin C/cisplatin and NaHCO_3_/cisplatin-treated mice were similar (*P* = 1.000), suggesting that both vitamin C and NaHCO_3_ pretreatment prevented cisplatin-induced increases in kidney weight ratios.

One out of 11 mice (9.09%) died (on day 20) in the saline/cisplatin group that precluded further cisplatin dosing. By contrast, mortality was absent in cisplatin-treated groups pretreated with vitamin C, NaHCO_3_ or resveratrol (n = 9–11 per group).

### mRNA Quantification of IL-1β, IL-6, IL-10 and TNFα in lumbar spinal cord and kidneys

**Figure 4:**
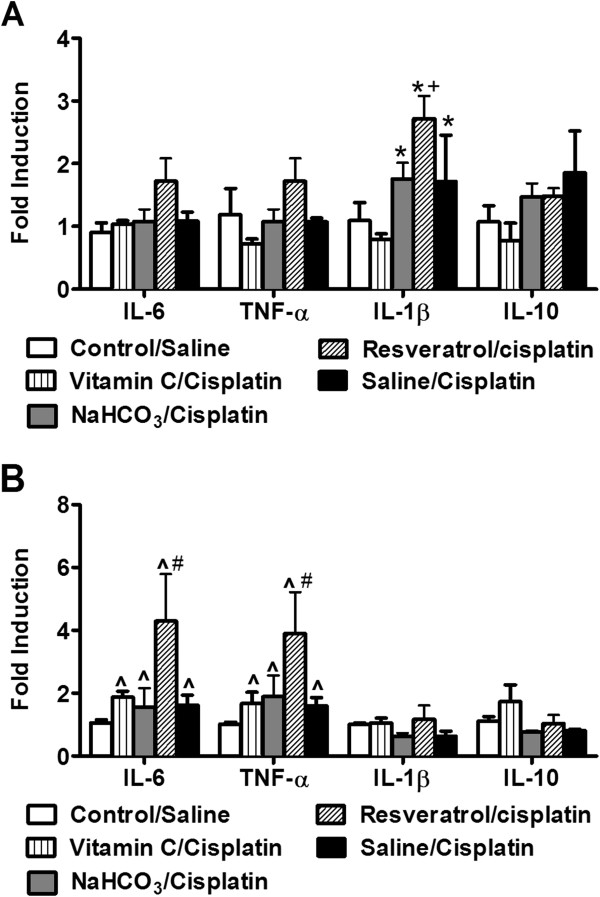
**mRNA levels of inflammatory cytokines (IL-1β, IL-6, IL-10 and TNFα) in lumbar spinal cord and kidneys.** IL-1β **(A)** mRNA levels are increased in lumbar spinal cord of saline, NaHCO_3_ or resveratrol/cisplatin-treated groups relative to the control/saline or vitamin C/cisplatin groups. Resveratrol/cisplatin group exhibited higher IL-1β mRNA levels relative to vitamin C/cisplatin group **(A)**. IL-6, TNFα and IL-10 spinal cord mRNA levels did not differ reliably between groups. **(B)** In the kidneys, levels of IL-6 and TNFα mRNAs were higher in cisplatin-treated mice compared to the control/saline group. Resveratrol/cisplatin group shows higher IL-6 and TNFα mRNA levels relative to control/saline group. IL-1β and IL-10 mRNA levels in the kidneys did not differ reliably between groups **(B)**. Data are expressed as mean ± s.e.m. (n = 4 per group). *P < 0.041 vs. control/saline or vitamin C/cisplatin (ANOVA, Bonferroni post hoc); + *P* < 0.046 for resveratrol/cisplatin group vs. vitamin C/cisplatin-treated group (ANOVA, Bonferroni post hoc); ^ *P* < 0.045 vs. control/saline group (ANOVA, Bonferroni post hoc); # *P* < 0.043 for resveratrol/cisplatin group vs. control saline group (ANOVA, Bonferroni post hoc).

RT-PCR analysis revealed that IL-1β mRNA levels were increased in the lumbar spinal cord of saline, NaHCO_3_ and resveratrol/cisplatin-treated groups (F_4,15_ = 3.27, *P* < 0.041) relative to control/saline or vitamin C/cisplatin groups (Figure 4A). Indeed, control/saline and vitamin C/cisplatin groups showed similar IL-1β mRNA levels in spinal cord (*P* = 1.000). The resveratrol/cisplatin group exhibited increased IL-1β mRNA levels relative to vitamin C/cisplatin (*P* < 0.046) group. By contrast, mRNA levels of IL-6 (*P* = 0.101), TNFα (*P* = 0.177) and IL-10 (*P* = 0.307) did not differ reliably between groups in the spinal cord (Figure 4A).

In the kidneys, cisplatin-treated mice exhibit higher mRNA levels of IL-6 (F_4,13_ = 3.81, *P* < 0.029) and TNFα (F_4,13_ = 3.29, *P* < 0.045) in comparison to the control/saline group (Figure 4B). Moreover, resveratrol/cisplatin group exhibited increased IL-6 (*P* < 0.03) and TNFα (*P* < 0.043) mRNA levels relative to the control/saline group (Figure 4B). mRNA levels of IL-1β (*P* = 0.415) and IL-10 (*P* = 0.153) in the kidneys did not differ reliably between groups (Figure 4B).

### Long term effects of vitamin C pretreatment on body weight in cisplatin-treated mice

**Figure 5:**
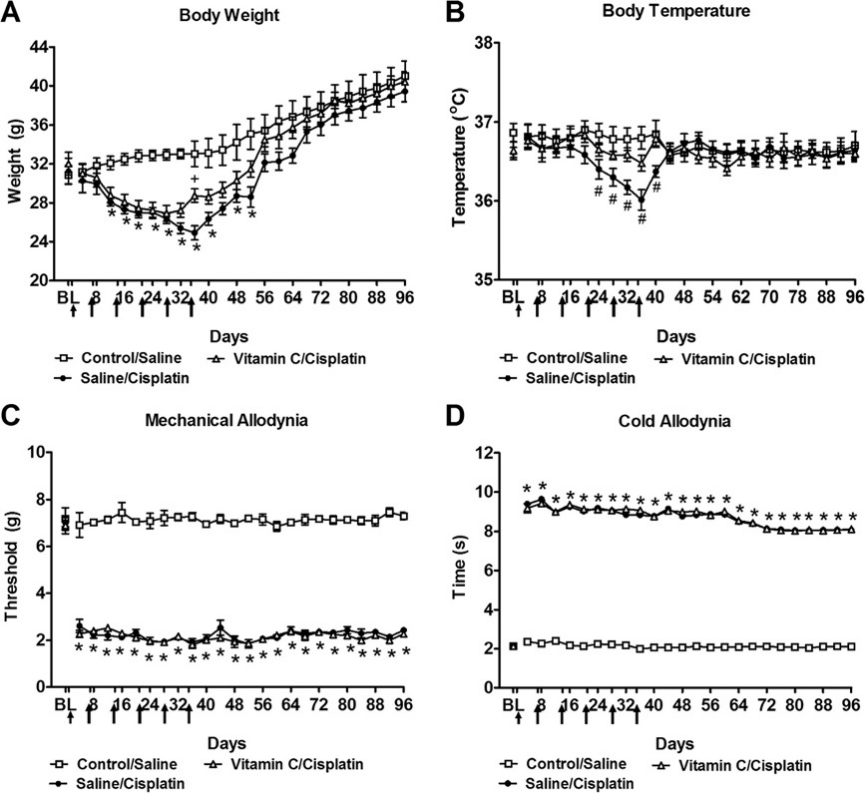
**Long term effects of cisplatin on body weight, body temperature, mechanical and cold allodynia in control/saline, saline/cisplatin or vitamin C/cisplatin groups.** Weight gain **(A)** is decreased in cisplatin-treated mice from day 12 to day 52 relative to control saline group. On day 36, saline/cisplatin exhibit lower body temperature than vitamin C/cisplatin group. Weight gain is similar in saline or cisplatin-treated groups from day 56 to 96. Body temperature **(B)** did not differ in the control/saline group relative to vitamin C/cisplatin group at any observation interval (from day 0 to day 96). Indeed,vitamin C pretreatment blunted cisplatin-induced decreases in body temperature. Time course of development of mechanical **(C)** and cold **(D)** allodynia in cisplatin-treated groups pretreated with saline or vitamin C. Arrows show timing of injections of chemotherapeutic agents. Data are expressed as mean ± s.e.m. (n = 6–8 per group). *P < 0.03 vs. control/saline group (ANOVA, Bonferroni post hoc); + *P* < 0.018 for vitamin C/cisplatin vs. saline/cisplatin group (ANOVA, Bonferroni post hoc); # *P* < 0.041 vs. control/saline or vitamin C/cisplatin-treated groups (ANOVA, Bonferroni post hoc).

Body weight gain did not differ in control/saline-treated mice in comparison to saline/cisplatin or vitamin C/cisplatin treatments from day 0 (Baseline) through day 8 (*P* ≥ 0.449 for each timepoint) or from day 56 to the end of the study (*P* ≥ 0.203 for each timepoint). By contrast, the control/saline group exhibited time-dependent increases in body weight (F_48,432_ = 5.78 *P* < 0.0001; Figure 5A) from day 12 to day 52 (*P* < 0.013 for all time points). Body weight was higher in control/saline compared to saline/cisplatin or vitamin C/cisplatin-treated groups (F_2,18_ = 4.27, *P* < 0.030). Indeed, weight gain in the control/saline group appeared on day 12 (*P* < 0.002) and persisted through day 52 (*P* < 0.002) (Figure 5A). Body weight did not differ between saline/cisplatin and vitamin C/cisplatin groups from day 12 to day 52 (*P* > 0.162) with one exception (i.e. day 36). Indeed, on day 36, body weight was lower in the saline/cisplatin group in comparison to vitamin C/cisplatin treatment (*P* < 0.018).

### Long term effects of vitamin C pretreatment on body temperature in cisplatin-treated mice

Body temperature did not differ in control/saline mice relative to other pretreatments (saline or vitamin C)/cisplatin in mice from day 0 (Baseline) (*P* = 0.211) to day 20 (*P* = 0.129) or from day 44 (*P* = 0.961) to day 96 (*P* = 0.861) (Figure 5B). However, body temperature was lower in the saline/cisplatin group relative to control/saline or vitamin C/ cisplatin groups from day 24 to day 40 (F_2,18_ = 5.04, *P* < 0.018; *P* < 0.041 for each time point; Figure 5B). Body temperature did not differ in the control/saline group relative to vitamin C/cisplatin group (*P* > 0.109) at any observation interval (from day 0 to day 96).

### Long term effects of vitamin C pretreatment on cisplatin-induced mechanical and cold allodynia

Both saline/cisplatin and vitamin C/cisplatin-treated groups exhibited lowered paw withdrawal thresholds relative to control/saline group (F_2,18_ = 3418.38, *P* < 0.0001) (Figure 5C), consistent with the cisplatin-induced development of mechanical allodynia. Mechanical allodynia was present from day 4 to day 96 (*P* < 0.0001) (Figure 5C) post initial cisplatin dosing. Furthermore, all cisplatin-treated groups showed similar increases in the duration of responding to acetone in comparison to control/saline group (F_2,18_ = 56173.62, *P* < 0.0001) (Figure 5D), consistent with development of cold allodynia. Cold allodynia was similarly present from day 4 to day 96 (*P* < 0.0001) (Figure 5D) post initial cisplatin dosing.

### Long term effects of vitamin C pretreatment on kidney functions in cisplatin-treated mice

**Figure 6:**
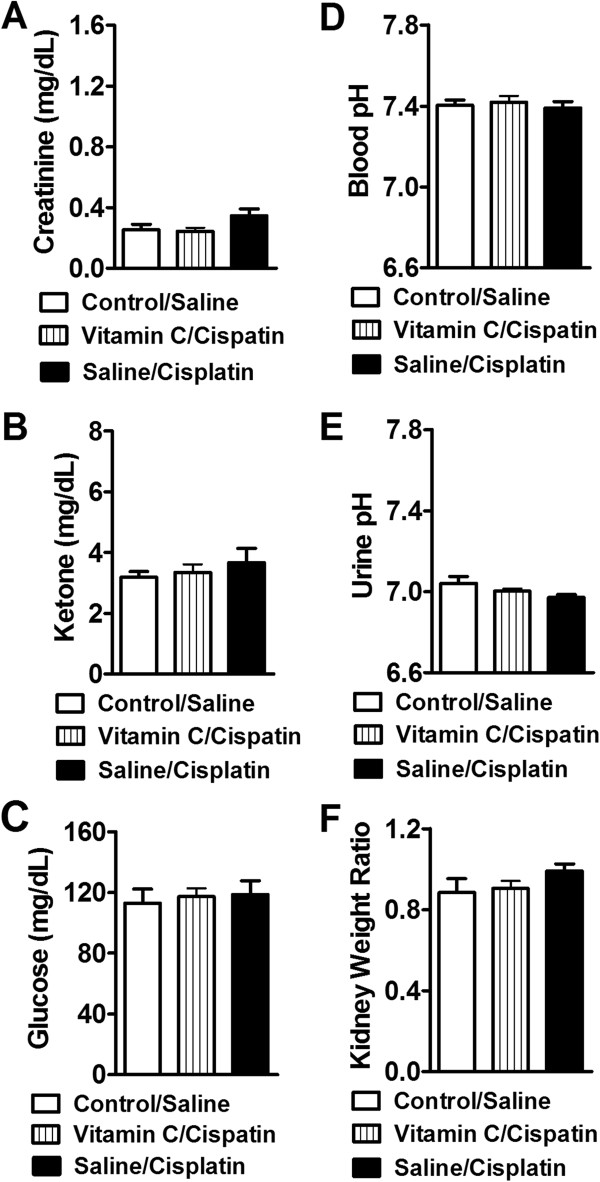
**Long term assessment of kidney functions in control/saline, saline/cisplatin or vitamin C/cisplatin groups.** Creatinine **(A)**, ketone **(B)** and glucose **(C)** levels as well as blood pH **(D)**, urine pH **(E)** and kidney weight ratios **(F)** were similar in control/saline, saline/cisplatin or vitamin C/cisplatin groups 60 days after the last cisplatin injection. Data are expressed as mean ± s.e.m. (n = 6–8 per group).

Creatinine (F_2,18_ = 2.63, *P* = 0.10), ketone (F_2,18_ = 0.55, *P* = 0.585) and glucose (F_2,18_ = 0.12, *P* = 0.884) levels were similar in control/saline, saline/cisplatin and vitamin C/cisplatin groups that received their last cisplatin treatment 60 days ago (Figure 6A-C). Furthermore, urine pH (F_2,18_ = 2.82, *P* = 0.086), blood pH (F_2,18_ = 0.236, *P* = 0.792) and kidney weight ratio (F_2,18_ = 0.32, *P* = 0.267) did not differ between control/saline, saline/cisplatin or vitamin C/cisplatin groups at this time point (Figure 6D-F). Thus, detrimental effects of cisplatin on kidney functions were no longer present 60 days following termination of cisplatin dosing in any group.

### Long term effects of vitamin C pretreatment on mRNA levels of cytokines (IL-1β, IL-6, IL-10 and TNFα) in the spinal cord and kidneys following cisplatin treatment

**Figure 7:**
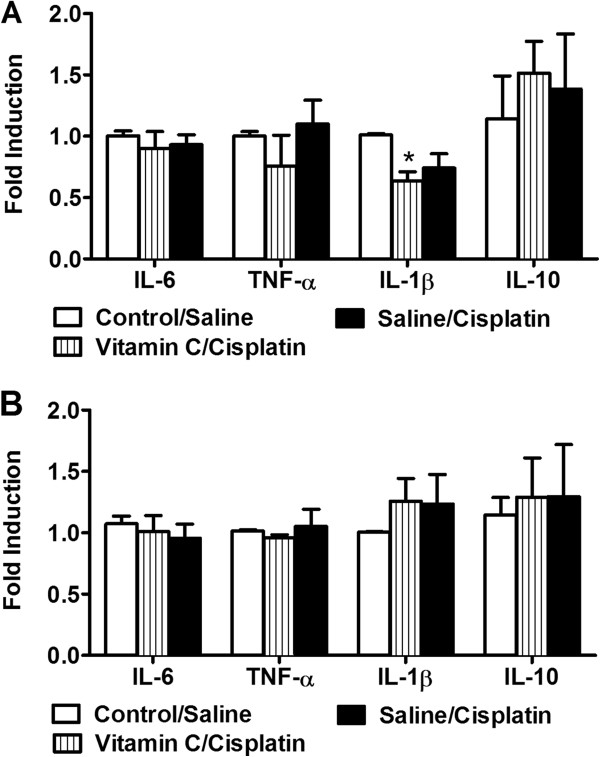
**Long term effects on mRNA levels of inflammatory cytokines (IL-1β, IL-6, IL-10 and TNFα) in the lumbar spinal cord and kidneys.** IL-1β mRNA levels are decreased in vitamin C/cisplatin treated group relative to control/saline group in the lumbar spinal cord **(A)**. IL-1β mRNA levels in spinal cord were similar in control/saline and saline/cisplatin groups. IL-6, TNFα or IL-10 mRNA levels were not altered in the spinal cord of control/saline, saline/cisplatin or vitamin C/cisplatin groups **(A)**. No changes were observed in mRNA levels of TNFα, IL-6, IL-1β and IL-10 in the kidneys **(B)** of control/saline, saline/cisplatin or vitamin C/cisplatin groups. Tissue was harvested 60 days following the last cisplatin dose. Data are expressed as mean ± s.e.m. (n = 4 per group). *P < 0.024 vs. control/saline group (ANOVA, Bonferroni post hoc).

Cisplatin mice pretreated with vitamin C exhibited modest decreases (F_2,9_ = 5.802, *P* < 0.024) in IL-1β mRNA in the lumbar spinal cord (Figure 7A) 60 days after the last cycle of cisplatin. IL-1β mRNA levels were higher in the control/saline group relative to vitamin C/cisplatin group (*P* < 0.028) (Figure 7A). IL-1β mRNA levels in the spinal cord were similar (*P* = 0.121) in control/saline and saline/cisplatin groups. No changes in IL-6 (*P* = 0.750), TNFα (*P* = 0.439) or IL-10 (*P* = 0.766) mRNA levels were observed in any group 60 days after the last cisplatin administration (Figure 7A).

No changes in mRNA levels of IL-6 (*P* = 0.749), TNFα (*P* = 0.750), IL-1β (*P* = 0.553) and IL-10 (*P* = 0.803) were detected in the kidneys (Figure 7B) of control/saline, saline/cisplatin or vitamin C/cisplatin groups at this time point.

## Discussion

Cisplatin produces nephrotoxicity that becomes more severe following repeated dosing and can result in mortality [6, 24]. Antioxidants (vitamin C and E, resveratrol, selenium, cysteine, quercetin) have been shown to protect against cisplatin-induced nephrotoxicity [20–23]. This effect is mediated by reducing oxidative stress which is protective against various injuries [21]. Vitamin C also attenuates the decrease in glomerular filtration rate that is induced by cisplatin treatment [21, 22]. Vitamin C and E also have hepatoprotective effects [25, 26]. Thus, sodium bicarbonate, resveratrol as well as vitamin C may exert protective effects on the kidney functions [8, 20, 25].

In the present study, we compared the effects of three different potentially beneficial pretreatments — sodium bicarbonate (4% NaHCO_3_ s.c_._), vitamin C (25 mg/kg s.c_._), and resveratrol (25 mg/kg s.c_._) — with saline (0.9% NaCl)) on cisplatin-induced kidney function, mRNA expression levels of pro-inflammatory cytokines, neuropathic allodynia and general health status. Our study demonstrates that cisplatin-treated mice receiving saline pretreatment exhibited elevated ketone, creatinine and kidney weight ratios, representative of nephrotoxicity. Vitamin C and sodium bicarbonate pretreatments lowered creatinine/ketone levels and restored kidney weight ratio whereas resveratrol pretreatment exhibited detrimental increases in both creatinine levels and kidney weight ratio that were similar to saline pretreatment. Thus, resveratrol pretreatment was inferior to either vitamin C or sodium bicarbonate for protecting against cisplatin-induced nephrotoxicity. However, all pretreatments decreased ketone levels compared to saline pretreatment. In our previous work in rats, pretreatment with 4% sodium bicarbonate (NaHCO_3_) in lieu of 0.9% saline (s.c.) prior to cisplatin dosing (3 mg/kg i.p. weekly up to 5 weeks) also showed beneficial effects on both kidney functions and general health status [8].

Weight loss was observed in all cisplatin-treated groups beginning on day 12, but was most pronounced in the resveratrol-pretreated group. The decrease in bodyweight was cisplatin-dependent because it was observed after the third and fourth cycles of cisplatin dosing. Interestingly, vitamin C-pretreated mice receiving cisplatin exhibited increases in body weight compared to the resveratrol-pretreated group, suggesting antioxidant superiority of vitamin C. In a long term study, vitamin C pretreatment was associated with increased body weight relative to saline/cisplatin treatment that normalized to control/saline animals by 56 days following initial cisplatin dosing.

Resveratrol and saline/cisplatin-treated mice also exhibited lowered body temperature (between days 20 to day 36) indicative of impaired health status. Only sodium bicarbonate and vitamin C pretreatments attenuated cisplatin-induced decreases in body temperature. By day 44 following initiation of cisplatin dosing (i.e. 9 days following cessation of cisplatin administration), cisplatin induced reductions in body temperature were no longer observed. Although cisplatin lowered body temperature, the values remain between 36 °C and 37 °C, so hypothermia (below 35 °C) was not observed in any condition [27]. Mice receiving cisplatin in the absence of anti-oxidant pretreatment could nonetheless be more susceptible to hypothermic effects of various pharmacological interventions [24].

However, none of the anti-oxidant pretreatments used altered the development or maintenance of neuropathic allodynia in our cisplatin dosing paradigm. Development of neuropathy was similar in the different pretreatment (sodium bicarbonate, vitamin C, resveratrol, or saline) groups receiving the same doses of cisplatin. These observations are consistent with the fact that mRNA expression levels of pro-inflammatory cytokines, though blunted by antioxidant treatment in kidney, remained elevated in lumbar spinal cord. Cisplatin-induced mechanical and cold allodynia was similarly preserved following pretreatment with sodium bicarbonate in rats [8, 28]. Moreover, mechanical and cold allodynia is maintained for at least 60 days following the last cisplatin treatment. Furthermore, morphine (6 mg/kg i.p.) and ibuprofen (6 mg/kg i.p.) both reversed mechanical and cold allodynia equivalently in all the pretreatment groups. To our knowledge, this is the first report of an anti-allodynic effect of ibuprofen in the cisplatin model. The impact of ibuprofen in altering the therapeutic activity of chemotherapeutic agents or radiation treatment has been previously evaluated [29–31] and shown to increase cellular sensitivity to radiation [30].

We further evaluated the ability of different antioxidant pretreatments to reduce the nephrotoxic effects of cisplatin, as documented by cisplatin-induced increases in creatinine levels, kidney weight ratio [8, 27, 32] and decreases in urinary pH [8, 19]. Indeed, resveratrol pretreatement failed to protect against several signs of nephrotoxicity in our study because cisplatin-induced increase in creatinine levels and kidney weight ratio were observed following resveratrol pretreatment in our study. Reports of resveratrol-induced protection against nephrotoxicity using a shorter duration of cisplatin (single administration of both cisplatin 5 mg/kg i.p. and resveratrol 25 mg/kg i.p. with survival time of 2 or 5 days post injection vs. 36 days in our study) treatment have been demonstrated [20]. Thus, beneficial effects of resveratrol may only be observed when cisplatin is administered acutely and short survival times are employed. Consequently, such beneficial effects may not translate to reductions in nephrotoxicity in the clinical situation where longer durations of cisplatin exposure are encountered. By contrast, cisplatin-treated groups receiving either vitamin C or sodium bicarbonate pretreatments exhibited lower creatinine levels and kidney weight ratios that did not differ from the control/saline group. Interestingly, only the sodium bicarbonate pretreatment showed elevated urinary pH (normal urine pH around 7.00) relative to the vitamin C or saline pretreated/cisplatin groups. This finding confirms results of previous studies in patients [19] and rats [8]. Indeed, an alkali pH (remain above 7.5) was observed in all (n = 26) patients pre-treated with 8.4% sodium bicarbonate before methotrexate treatment [19]. Resveratrol-treatment was associated with a urinary pH that is similar to sodium bicarbonate pretreatment, likely due to antioxidants properties [20]. All pretreatments failed to alter blood pH, as observed previously following sodium bicarbonate pretreatment in rats [8] (Figure 3D). Following our long term evaluations of cisplatin treatment, creatinine, kidney weight ratio, urine and blood pH were all normalized to control levels in the different pretreatment groups (control/saline, saline or vitamin C/cisplatin) measured 60 days after the last cisplatin injection.

We also evaluated the impact of our pretreatments on fatty acid metabolism by measuring levels of ketone in whole blood. Ketone is an end product of fatty acid metabolism and high ketone levels are indicative of keto-acidosis, a serious medical condition characterized by high acidity of bodily fluids [27, 33, 34]. Cisplatin increased ketone levels in saline pretreated groups in the absence of antioxidant treatment (Figure 3B) although normal (7.37 ± 0.04) blood pH (normal blood pH between 7.35-7.45) [27] was observed. Thus, all pretreatments effectively blunted the cisplatin-induced increase in ketone levels. However, cisplatin-induced elevations in ketone levels were normalized by 60 days following cessation of cisplatin dosing. Glucose levels (50–135 mg/dl) did not differ amongst any of the saline or cisplatin treated groups. Thus, our induction of neuropathy in our cisplatin dosing paradigm cannot be associated with high levels of glucose found in models of diabetic neuropathy [35].

In the spinal cord, cisplatin produced long term elevation in mRNA levels of the cytokine IL-1β in the saline, NaHCO_3_ or resveratrol/cisplatin-treated groups, with resveratrol/cisplatin showing higher IL-1β mRNA levels relative to vitamin C/cisplatin group. The observed increase in IL-1β mRNA is consistent with increases in IL-1β protein following acute treatment with high dose (30 mg/kg i.p.) of cisplatin [36] or in *in vitro* studies using different cell lines [37, 38]. IL-1β has been colocalized to astroglia but not neurons or microglia [39]. At 60 days post cisplatin, IL-1β mRNA levels are decreased in the vitamin C/cisplatin group in comparison to the control/saline group whereas no changes are observed in IL-6, TNF-α and IL-10 mRNA levels. This neuroprotective effect of vitamin C observed here is consistent with results from previous studies [40, 41].

In the kidneys, cisplatin increased both IL-6 and TNF-alpha mRNAs in all pretreatment groups relative to the control/saline group (Figure 4B). Increases in kidney IL-6 protein levels have been reported following cisplatin treatment in an acute renal failure model [36]. Cisplatin-induced increases in TNF-α protein expression has also been observed *in vitro*[37, 38] or following acute administration of cisplatin in mice (30 mg/kg i.p., [42]: 15 mg/kg i.p., [43]). However, 60 days after the last of six cisplatin treatments, IL-6, TNF-α, IL-1β and IL-10 mRNA levels in the kidneys are similar in all treatment groups. Albumin-thioredoxin [43] or rhodobacter sphaeroides (Lycogen) in mice treated acutely with cisplatin [42] also blunts the elevation of IL-6 and TNF-α mRNA levels in the kidney. More work is necessary to show that inhibition of IL-6 and TNF-α mRNA levels in the kidney by antioxidants mediates renal protective effects following repeated cisplatin injections and long term nephroprotective effects. Indeed, to our knowledge, the present study is the first to evaluate the long term beneficial effects (60 days after the last of six cisplatin treatments) of antioxidant pretreatments (with vitamin C) on body weight, body temperature, kidney functions and interleukins in mice receiving repeated cycles of cisplatin (once a week 5 mg/kg i.p. for 6 weeks).

## Conclusions

Our studies provide direct evidence that once weekly subcutaneous injections of either sodium bicarbonate (NaHCO_3_) or vitamin C have a beneficial impact on animal health status and kidney function (normal creatinine levels, normal kidney weight ratio and absence of mortality) following repeated cisplatin treatment. Because development of chemotherapy-induced neuropathy is not altered by anti-oxidant treatments, our methods permit long term assessments of cisplatin-induced neuropathy (i.e. at least 60 days following the last of six once weekly cisplatin injections across 96 days) that are not confounded by unacceptable toxicity or impairment in animal health. Furthermore, a full reversal of cisplatin-induced mechanical and cold allodynia was produced by either morphine or ibuprofen treatment and efficacy did not differ as a function of the various pretreatment conditions (i.e. sodium bicarbonate, vitamin C, resveratrol or saline). By contrast, both saline or resveratrol pretreatments prior to cisplatin negatively impacted renal function. Moreover, IL-1β mRNA levels are increased in the lumbar spinal cord of saline, NaHCO_3_ and resveratrol/cisplatin-treated groups whereas IL-6 and TNF-alpha mRNAs are elevated in the kidneys in all cisplatin-treated groups, but normalized by 60 days after the last cisplatin treatment. Thus, administration of sodium bicarbonate or vitamin C prior to cisplatin treatment has long lasting beneficial effects on general health of rodents. The present approach employing antioxidant treatment should permit comprehensive study of the different mechanisms underlying cisplatin-induced neuropathy and facilitate identification of effective prophylactic treatments.

## Methods

### Subjects

Male C57BL/J mice (Jackson labs, Bar Harbor, ME, USA) weighing 26–33 g before testing, were used. Animals were single housed in standard plastic cages with sawdust bedding in a climate-controlled room, under a 12 h light/dark cycle. The mice received free access to standard rodent chow and water. All experimental research protocols was carried out in accordance with the National Institute of Health Guidelines for the Care and Use of Laboratory Animals and protocols approved by the Bloomington Indiana University Institutional Animal Care and Use Committee and all procedures conformed to the guidelines for the treatment of animals established by the International Association for the Study of Pain [44].

### Drugs

Morphine sulfate and ibuprofen sodium salt were purchased from Sigma-Aldrich (St-Louis, MO, USA). Vitamin C (acid ascorbic), sodium bicarbonate (NaHCO_3_) and cisplatin were purchased from Tocris (Ellisville, MO, USA). Resveratrol was provided by InvivoGen (San Diego, CA, USA). Doses of morphine and ibuprofen were selected based upon efficacy demonstrated in previous studies [28, 45, 46]. Resveratrol was dissolved in 10% dimethylsulfoxide (DMSO) [20]. Vitamin C, sodium bicarbonate (NaHCO_3_), morphine and ibuprofen were dissolved in normal saline (0.9% NaCl in water) [22, 28, 46].

### Protocol

First, we measured body weight and body temperature of saline- (n = 18) or cisplatin-treated mice that were pretreated with either: saline, vitamin C, resveratrol or NaHCO_3_ (n = 9-11 per group). Using the same cohorts of mice, we evaluated the effects of saline- or cisplatin-treatments on mechanical paw withdrawal threshold (electro von Frey stimulation) and latency of paw withdrawal to acetone (cold responsiveness). A subset of these animals was used to evaluate kidney functions (creatinine levels in whole blood, urine and blood pH and kidney weight ratio) as well as quantification of mRNA levels of interleukins (IL-1β, IL-6, IL-10 and TNFα) in spinal cord and kidneys. Another cohort of mice was used to evaluate the anti-allodynic effects of morphine and ibuprofen on cisplatin-induced mechanical and cold allodynia in the different pretreatment (saline, vitamin C, resveratrol or NaHCO3) groups. Subsequent groups of mice treated with saline, saline/cisplatin and vitamin C/cisplatin were evaluated to establish the long term effects (60 days following termination of cisplatin dosing) of the last cisplatin weekly administration on body weight, body temperature, kidney functions, sensitivity to mechanical and cold stimulation as well as mRNA expression levels of pro-inflammatory cytokines (IL-1β, IL-6, IL-10 and TNFα) in spinal cord and kidneys.

### Temperature measurement

Rectal temperature was assessed in animals receiving different pretreatments (saline, bicarbonate (NaHCO_3_), vitamin C and resveratrol) using a rectal probe (Physitemp RET-2 rectal probe for rats, Clifton, NJ, USA) and meter (Physitemp Model BAT-12R, Clifton, NJ, USA). Cisplatin has previously been shown to induce aberrant changes such as lowered body temperature [6, 8, 29]. Body temperature was recorded every four days from day 0 to day 36. The same animals were used to evaluate body weight and body temperature as well as mechanical and cold allodynia. A subset (n = 8 per group) of these animals was used to evaluate kidney functions. For the long term study (60 days post termination of cisplatin dosing), another cohort of mice was used to measure and evaluate: body temperature, body weight, mechanical and cold allodynia.

### Development of neuropathic pain

Cisplatin (Tocris, Ellisville, MO, USA) was administered intraperitoneally (i.p.) once a week at a dose of 5 mg/kg for 6 (36 days) weeks (cumulative dose: 30 mg/kg i.p.) [5]. Cisplatin was diluted in normal saline (0.9% NaCl) and delivered in a volume of 10 ml/kg body weight. Control groups were injected with an equivalent volume of saline (i.p.) in lieu of cisplatin [8]. Before each cisplatin/saline i.p. injection either (1) 0.9% NaCl [15]; (2) 4% sodium bicarbonate (NaHCO_3_ dissolved in 0.9% NaCl) [8]; (3) vitamin C (25 mg/kg s.c_._) [22]; or (4) resveratrol (25 mg/kg s.c_._) [20] was administered subcutaneously in a final volume of 1 ml. Injections were always performed after completion of mechanical and cold withdrawal testing.

### Assessment of mechanical allodynia

Mechanical withdrawal thresholds were assessed using a digital Electrovonfrey Anesthesiometer (IITC Life Sciences, Woodland Hills, CA) equipped with a semi-flexible tip [28, 47]. The digital Electrovonfrey was used so the weight is constantly displayed. Mice were placed in individual plastic cages on an elevated wire mesh platform, and were allowed to habituate to the testing apparatus for at least 30 minutes until exploratory behavior was no longer observed. Force was applied to the midplantar region of each hindpaw in each study by a single experimenter. Stable baseline responses were obtained prior to experimental testing. Mechanical stimulation was terminated upon paw withdrawal; consequently, there was no upper threshold limit set for termination of a trial. Paw withdrawal thresholds were assessed in duplicate in each paw. Mechanical withdrawal thresholds were measured every 4 days over 36 days. Testing took place on days 0, 4, 8, 12, 16, 20, 24, 28, 32 and 36 for all animals. For the long term study (60 days post-cisplatin treatment), mice in relevant cohorts were also tested every 4 days from day 36 to 96.

### Assessment of cold allodynia

Cold allodynia was measured by applying drops of acetone to the plantar surface of the hind paw as previously described [47, 48]. Mice were placed in individual plastic cages on an elevated platform and were habituated for at least 30 min until exploratory behaviors ceased. Acetone was loaded into a one ml syringe barrel with no needle tip. Air bubbles were cleared from the syringe prior to acetone application. One drop of acetone (approximately 20 μl) was applied through the mesh platform onto the plantar surface of the hind paw. Care was taken to gently apply the bubble of acetone to the skin on the paw without inducing mechanical stimulation through contact of the syringe barrel with the paw. Paw withdrawal time in a 60 s observation period after acetone application was recorded. Paw withdrawal was sometimes associated with a secondary response with the animal, such as rapid flicking of the paw, chattering, biting, and/or licking of the paw. Testing order alternated between paws (i.e. right and left) until five measurements were taken for each paw. An interstimulation interval of approximately 5 minutes was allowed between testing of right and left paws. Cold allodynia testing took place on days 0, 4, 8, 12, 16, 20, 24, 28, 32 and 36 for all animals. For the long term study (60 days post-cisplatin treatment), the mice were also tested every 4 days from day 36 to 96.

### Assessment of kidney functions

Creatinine, ketone and glucose levels (mg/dL) were measured in whole blood using the PTS CardioChek diagnostic apparatus (Cliawaived.com, San Diego, CA, USA). Urine and blood were extracted post mortem [8]. Urine and blood pH was measured using a digital pH 110 m (Oakton Instruments, Vermon Hills, IL, USA). The kidney to body weight ratio was also measured [20].

### Sample preparation for RT-PCR analysis

Animals receiving control/saline (n = 4), saline/cisplatin (n = 4), sodium bicarbonate (NaHCO_3_)/cisplatin (n = 4), vitamin C/cisplatin (n = 4) and resveratrol (n = 4) (see methods) were killed by rapid decapitation without anesthesia at day 36 following initiation of cisplatin dosing to generate spinal cord and kidney samples used for mRNA quantification. Separate cohorts of mice treated with control/saline (n = 4), saline/cisplatin (n = 4) and vitamin C/cisplatin (n = 4) were killed by rapid decapitation without anesthesia at day 96 (60 days following termination of cisplatin dosing) to generate spinal cord and kidney samples used in mRNA quantification. Lumbar spinal cord and both kidneys were rapidly fast frozen in isopentane precooled on dry ice (-30°C) and stored at -80°C until use as described previously [28].

### Quantification of spinal cord and kidney mRNA

Real time RT-PCR was used to quantify mRNA levels as previously described [28]. RNA from spinal cord and kidneys of mice treated with control/saline, saline/cisplatin, NaHCO_3_/cisplatin, vitamin C/cisplatin and resveratrol/cisplatin were extracted using a TRIzol (Ambion, CA, USA)/RNeasy (Qiagen, CA, USA) hybrid protocol according to manufacturer’s instructions. Purified RNA from each sample was then treated with DNase 1(New England BioLabs, MA, USA). Expression levels of IL-1β, IL-6, IL-10 and TNFα mRNAs were quantified using one step RT-PCR with PowerSYBR green PCR kit (Applied Biosystems, Carlsbad, CA,USA) by a Matercycler ep realplex RT-PCR machine (Eppendorf Norh America Inc., Hauppauge, NY,USA). GAPDH (glyceraldehyde-3-phosphate dehydrogenase) was used as internal standard to normalize mRNA levels. Primers used were as follows: mouse GAPDH (sense: 5′-GGGAAGCTCACTGGCATGGC-3′, anti-sense: 5′- GGTCCACCACCCTGTTGCT-3′); mouse IL-1β (sense: 5′-CGTGGACCTTCCAGGATGAG-3′, anti-sense: 5′-CATCTCGGAGCCTGTAGTGC-3′); mouse IL-6 (sense: 5′-GCCTTCTTGGGACTGATGCT-3′, anti-sense: 5′-TGCCATTGCACAACTCTTTTC-3′); mouse IL-10 (sense: 5′-GGCGCTGTCATCGATTTCTC-3′, anti-sense: 5′-GGCCTTGTAGACACCTTGGTC-3′); mouse TNFα (sense: 5′-CGTCGTAGCAAACCACCAAG-3′, anti-sense: 5′-TAGCAAATCGGCTGACGGTG-3′).

### Statistical analysis

All experiments were conducted in a blinded manner. Animals were randomly assigned to experimental conditions. Paw withdrawal thresholds (mechanical) and latencies (cold) were calculated for each paw and averaged. Data were analyzed using analysis of variance (ANOVA) for repeated measures or one-way ANOVA as appropriate. The Greenhouse-Geisser correction was applied to all repeated factors; degrees of freedom reported for significant interactions are the uncorrected values. The source of significant interactions was further evaluated by performing one way ANOVAs at each individual time point, followed by Bonferroni post hoc tests. The different components of the total variation were settled *a priori* using multiple regression analysis [49]. Analyses were performed using SPSS statistical software (version 21.0; SPSS Incorporated, Chicago, IL, USA). *P* < 0.05 was considered significant.

## Abbreviations

ANOVA: Analysis of variance
BL: Baseline
cis: Cisplatin
NaCl: sodium chloride
NaHCO_3_: sodium bicarbonate
IL-1: Interleukin 1
IL-1b: Interleukine 1 beta
IL-10: Interleukin 10
inj: Inject
ip: intraperitoneal
sc: Subcutaneous
TNFα: tumor necrosis factor alpha.
